# Supplementary material for: BLASE: bulk linkage analysis for single cell experiments - teasing out the secrets of bulk transcriptomics with trajectory analysis
Source: BMC Methods. Author manuscript; Available in PMC 2026 Sep 23. (PMC7619496; doi:10.1186/s44330-026-00082-7)
Supplement: Supplementary file 1 [file EMS217646-supplement-Supplemental_figures_and_tables.pdf]

Supplement: BLASE: Bulk Linkage Analysis for Single Cell Experiments - Teasing Out the Secrets of Bulk Transcriptomics with Trajectory Analysis

A Supplemental Information

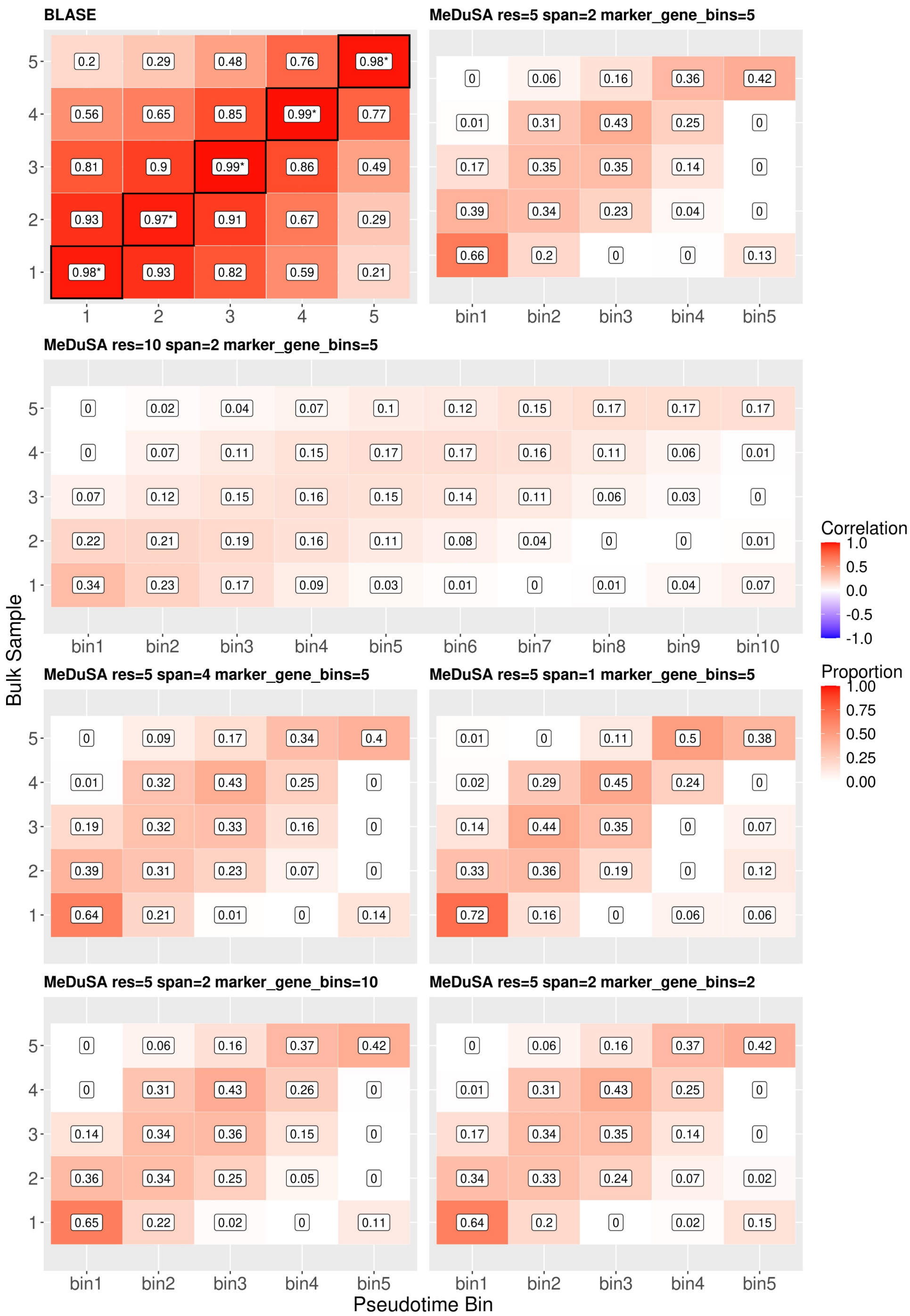

Fig. A1 Plots showing results of BLASE and MeDuSA with varying parameter choices from the *C. elegans* ciliated neurons dataset used to benchmark MeDuSA by its authors.

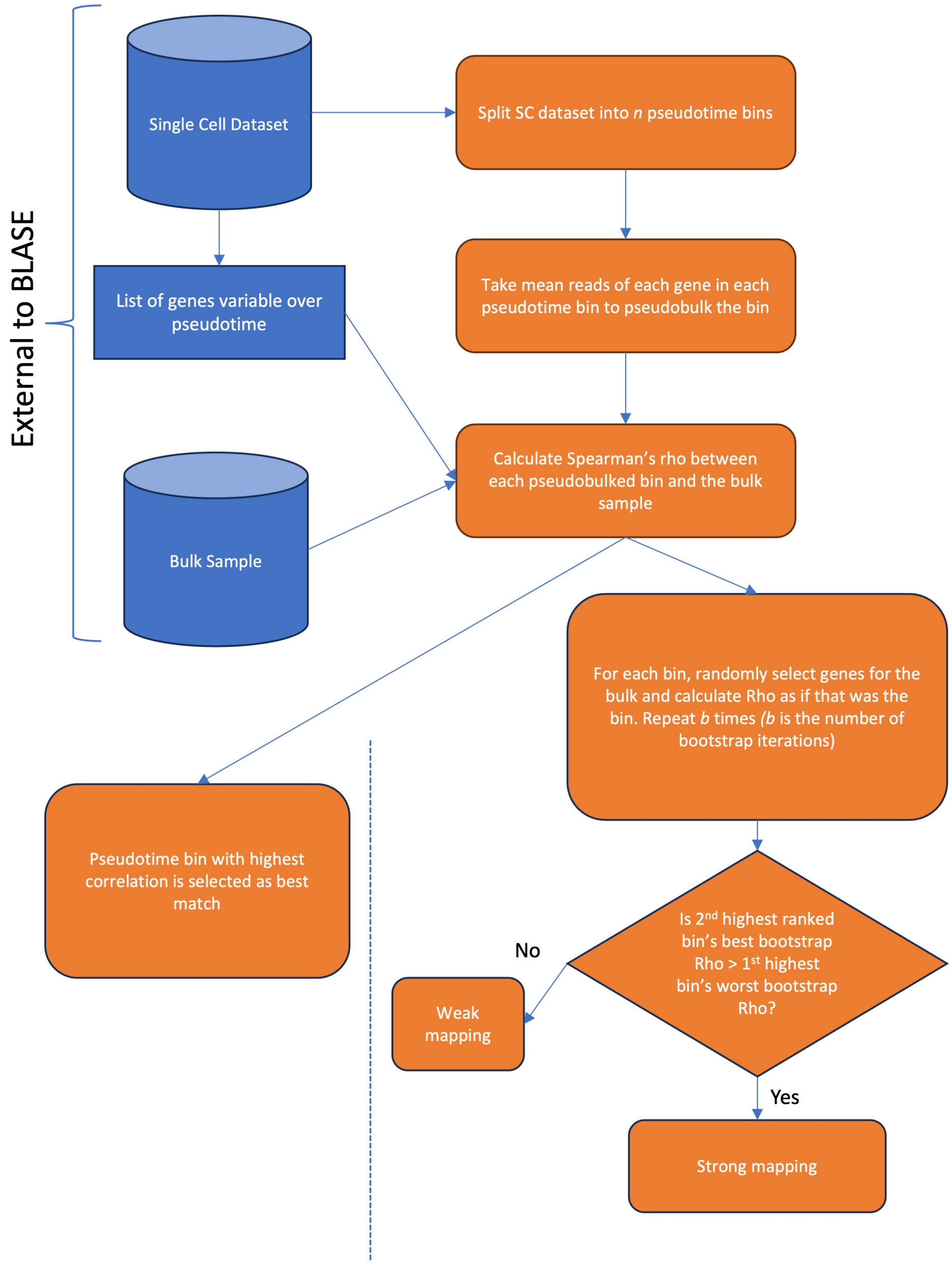

Fig. A2 Flowchart detailing the steps of the BLASE algorithm. Three inputs (scRNA-seq reference, list of genes that change over pseudotime, and a bulk RNA-seq sample to map) are shown in blue. Steps taken by BLASE are shown in orange.

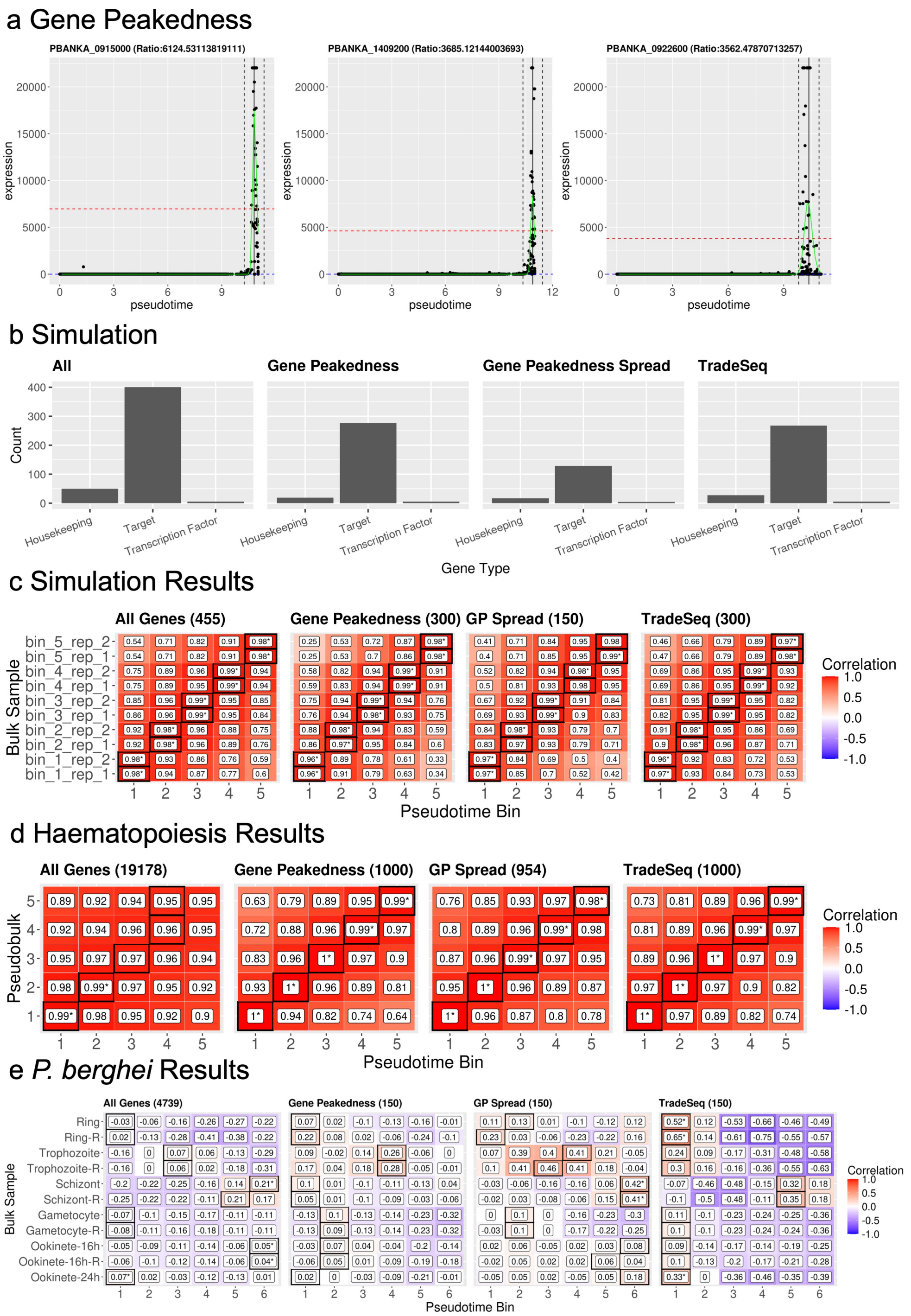

Fig. A3 Gene selection methods for BLASE a, The top three genes (sorted by descending BLASE gene peakedness) for the Howick et al. 2019 *P. berghei* reference dataset. Normalised counts are plotted as a scatter plot. Two dotted black lines show the 10% window around the peak, as calculated by the smoothed values from a GLM fitted to the gene. The dashed red line indicates the mean count within the window, and the dashed blue line the mean outside the window. b, Counts of the types of simulated gene in the simulated test dataset, for all genes, the genes selected by tradeSeq, the genes selected by BLASE gene peakedness and the genes selected by BLASE spread gene selection. These methods filter out housekeeping genes which are not informative about the trajectory. c,d,e, BLASE result heatmaps generated using genes selected by four methods (all genes, BLASE gene peakedness selection, gene peakedness spread, tradeSeq selection), for three scRNAseq references. c: Simulated dataset, d: Mende et al. 2020 haematopoiesis dataset, e: Howick et al. 2019 *P. berghei* dataset.

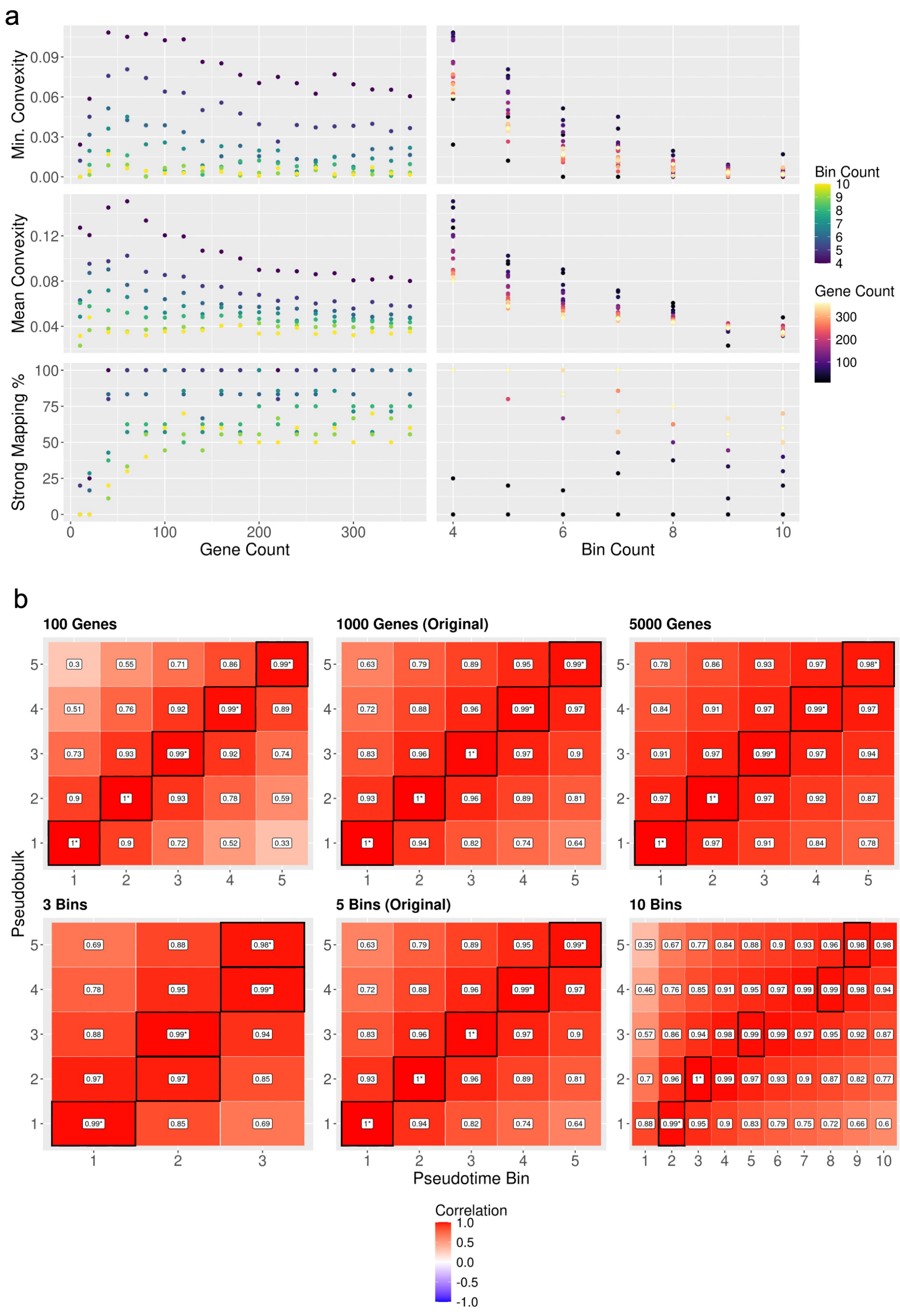

Fig. A4 a, BLASE Hyperparameter selection plot, created with `find_best_params_results()`. This plot shows the worst and mean "convexity" of a set of parameters. Convexity is calculated by pseudobulking each bin, and using BLASE to map them, and finding the distance between the best mapping (in the expected case, the bin the pseudobulked sample is generated from) and the bin with the second highest correlation. A higher "convexity" is better. The plots show the x-axis as either the number of genes used (left side) or the number of bins used (right side). Also shown is the percentage of strong mappings for each pair of n bins and genes. b, A set of BLASE result heatmaps showing the effect of varying numbers of genes and bins on the same dataset. Results generated from the Mende et al. 2020 haematopoiesis scRNA-seq dataset.

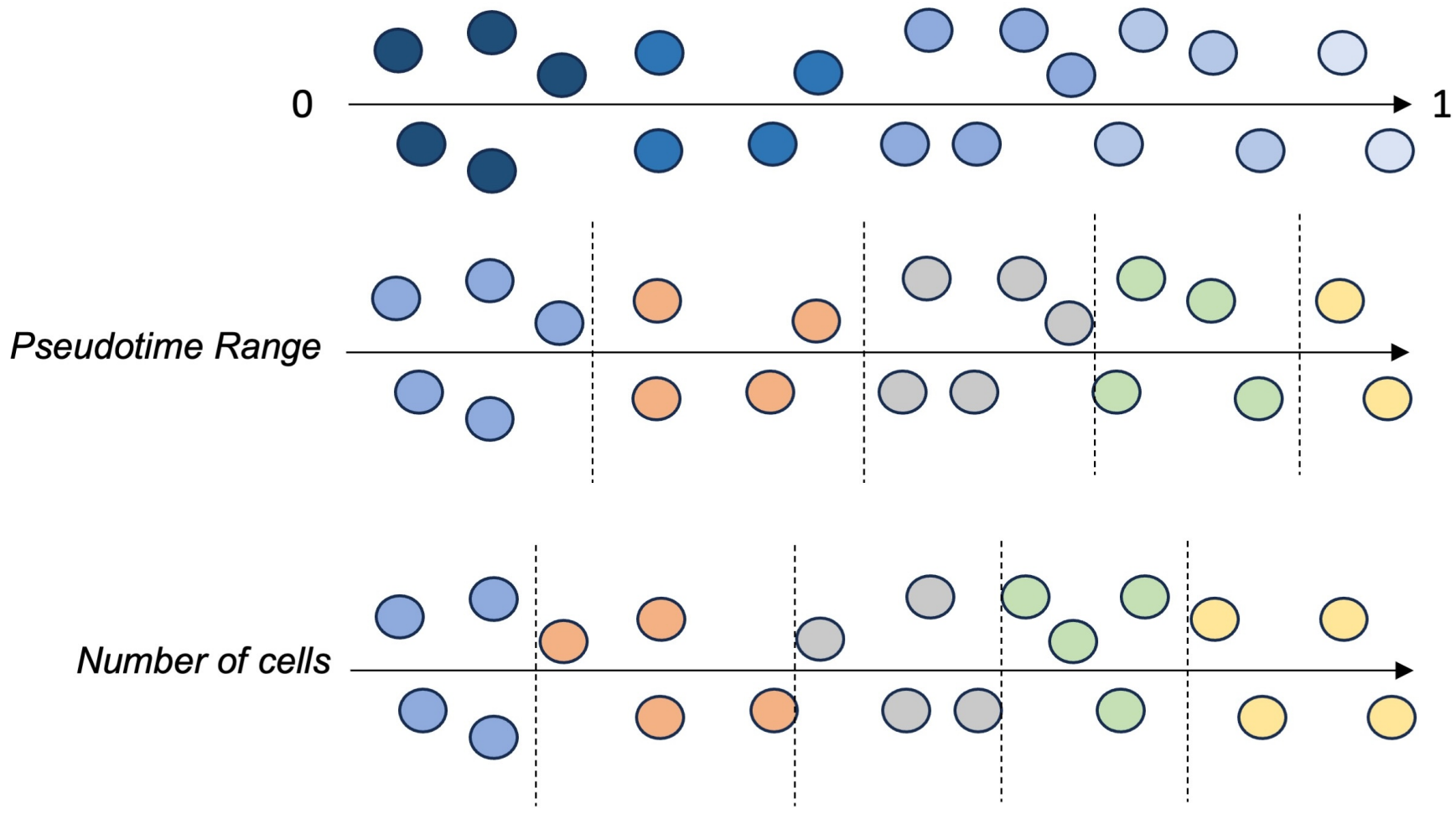

Fig. A5 Schematics of BLASE pseudotime bin selection methods. Pseudotime_range: ensures all bins have the same width of pseudotime (in this case, 0.2). This allows varying numbers of cells. This is the recommended method as it preserves a constant transcriptional "distance." Cells: ensures all bins have the same number of cells, allowing varying widths of pseudotime.

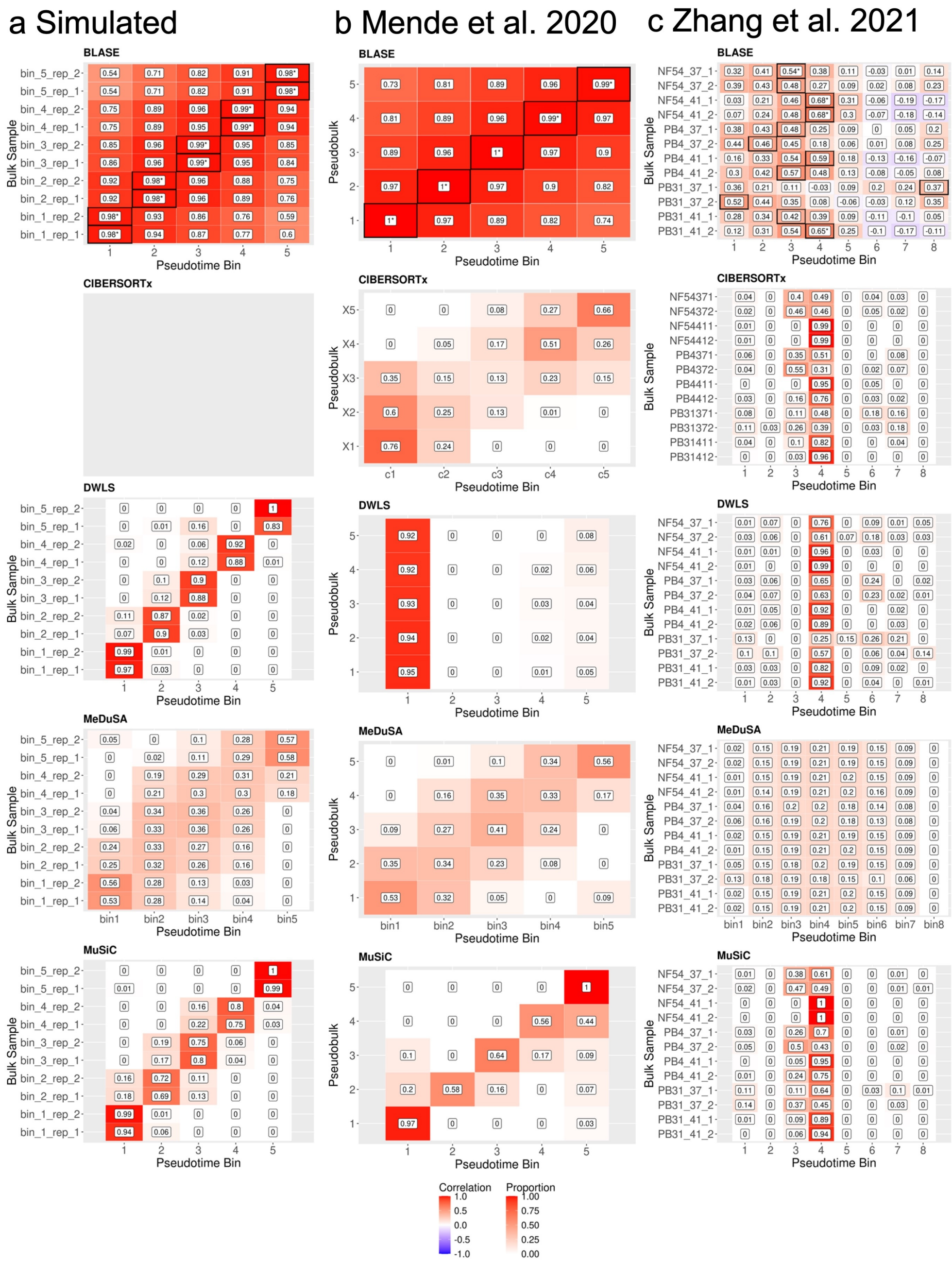

Fig. A6 BLASE performance compared with other tools on several datasets a, BLASE (All genes), CIBERSORTx, MuSiC, MeDuSA, and SCDC result heatmaps showing the predicted mappings on a simulated dataset (using a test/train split). b, The results from running the same tools as a on the Mende et al. 2020 haematopoiesis dataset (test/train split). BLASE used a TradeSeq generated genelist. c, The results from running the same tools as a on the Dogga et al. 2023 scRNA-seq with the Zhang et al. 2021 bulk RNA-seq data. BLASE used a Gene Peakedness gene selection.

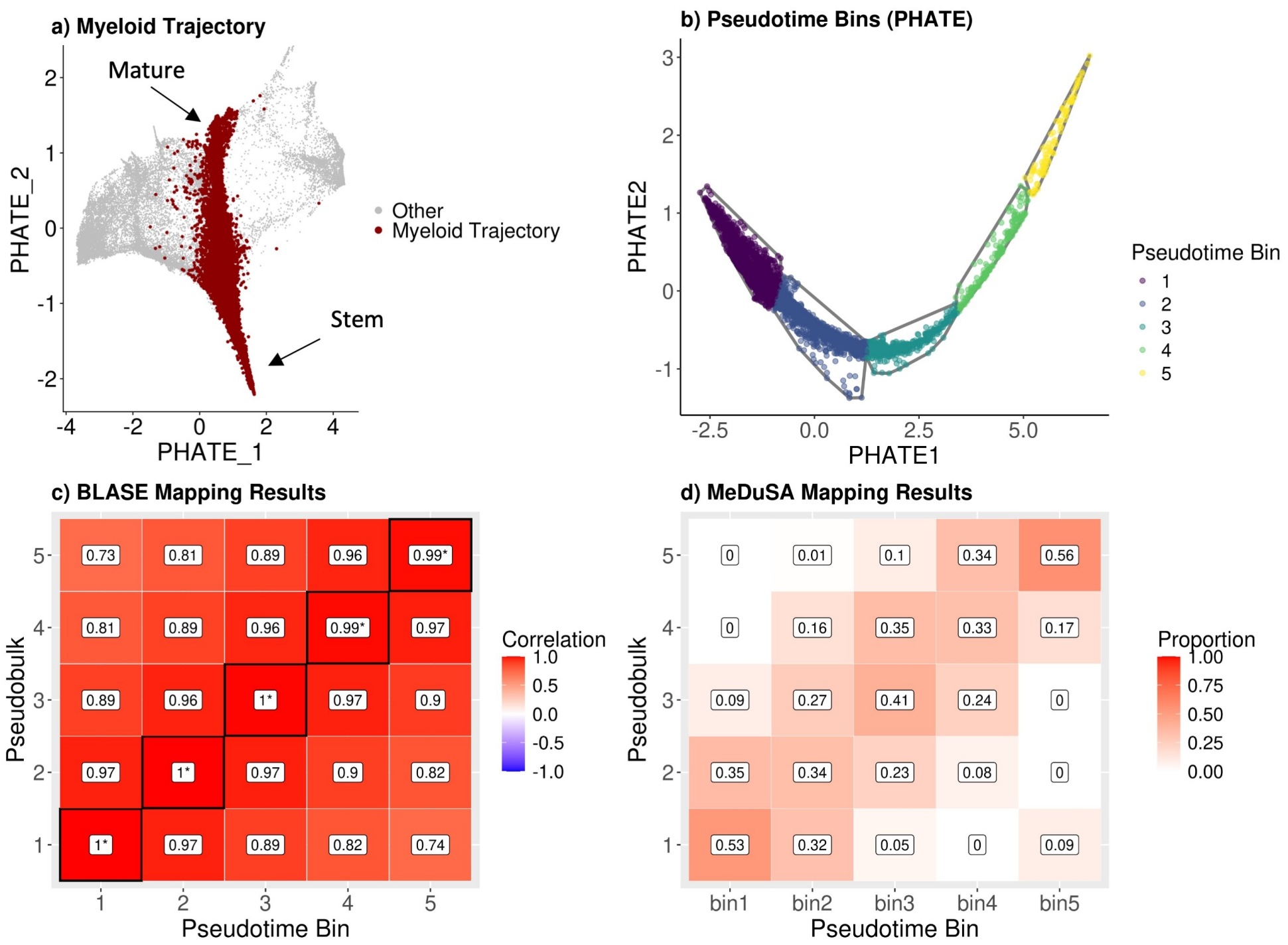

Fig. A7 Validating BLASE with a test/train pseudobulked dataset of haematopoiesis. a, The myeloid cell differentiation lineage selected for testing BLASE results, highlighted on all other cells in the PHATE projection. b, A PHATE embedded plot of the single-cell training dataset (including only the myeloid differentiation lineage) coloured by assigned pseudotime bins. c, A heatmap showing Spearman's Rho (of the gene list calculated by TradeSeq) between pseudotime bins and bulk RNA-seq samples. Asterisks indicate strong results, and black outlines indicate the best match. Pseudotime bins are shown on the x-axis, tested bulk samples are on the y-axis. d, The mapping proportions from MeDuSA deconvoluting the same data as c.

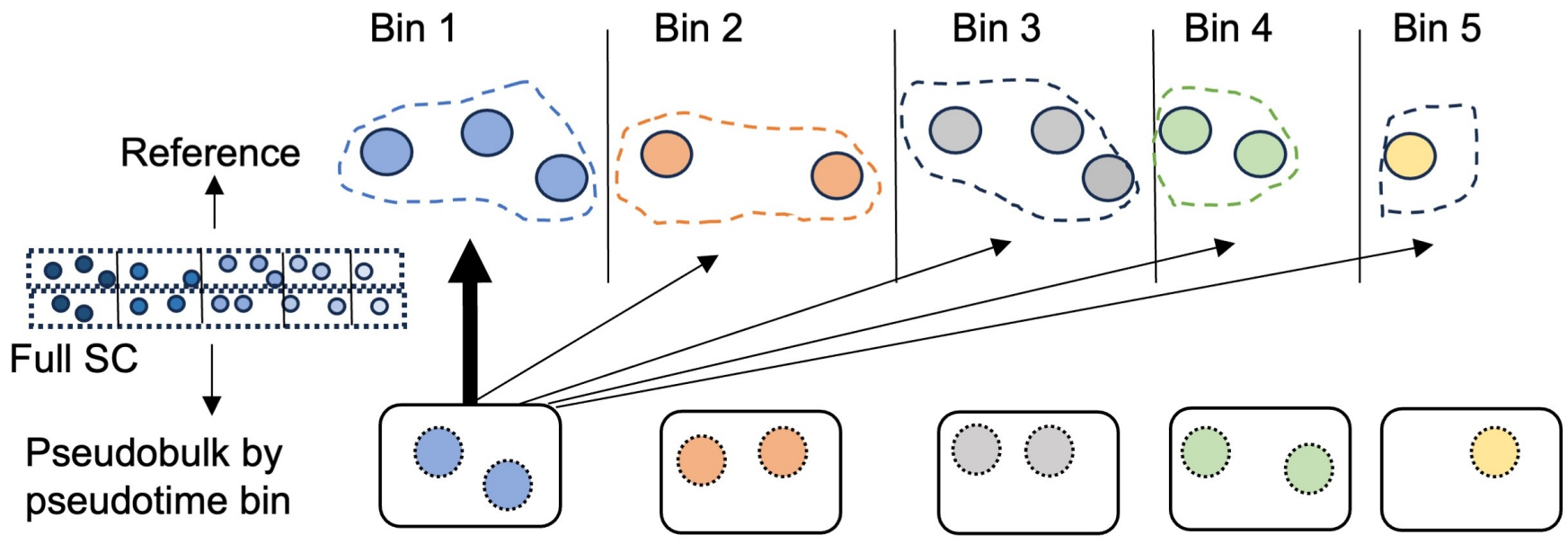

Fig. A8 Generation of testing datasets. A scRNA-seq dataset was generated, then randomly split into two groups. One group was used as reference, and the second group was pseudobulked by pseudotime bin.

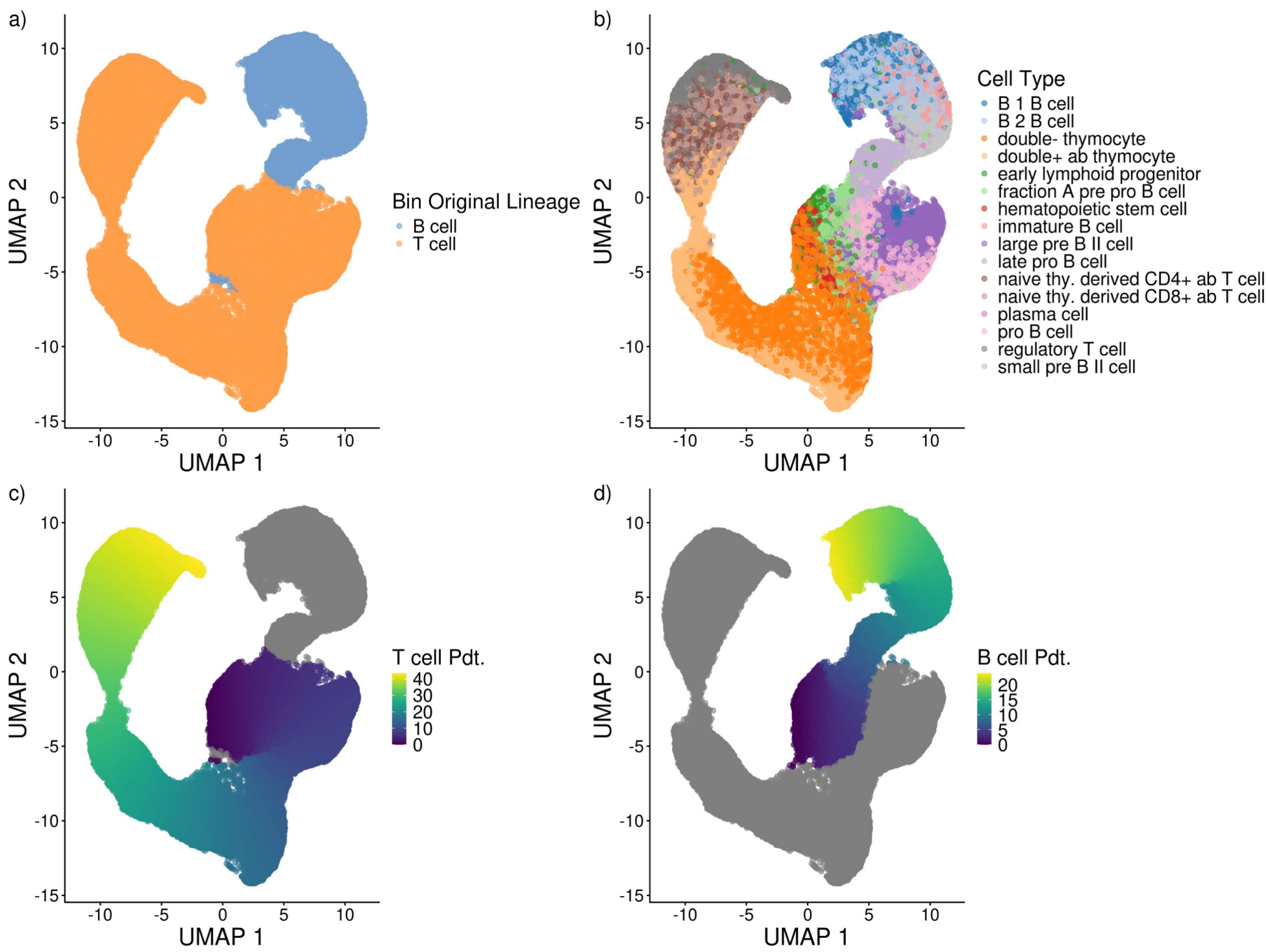

Fig. A9 UMAP projections of the scRNA-seq reference of lymphoid development, coloured by a, the original lineage used to generate the pseudotime bin a cell is a member of (see Methods for binning multiple trajectories), b, Cell type, c, the T cell pseudotime calculated by Slingshot, and d, the B cell pseudotime calculated by slingshot

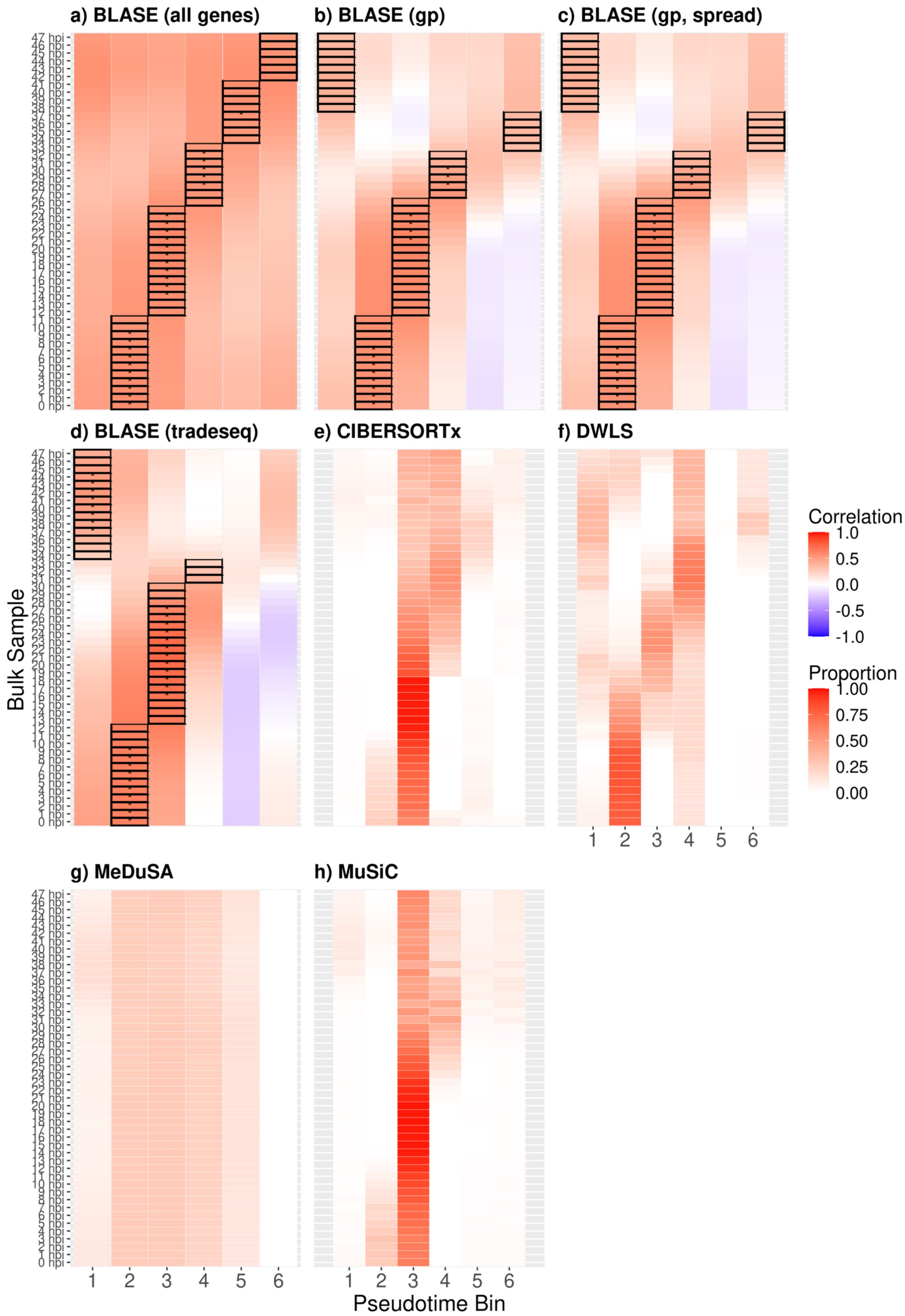

Fig. A10 BLASE gene selection methods compared with deconvolution packages (Painter et al. 2018 / Dogga et al. 2023) a, BLASE result heatmap showing the predicted mappings in the Dogga et al. 2023 scRNA-seq for bulk RNA-seq the from Painter et al. 2018, using all genes. b, BLASE result heatmap showing the same data as in a, when genes selected by the BLASE gene peakedness method. c, BLASE result heatmap showing the same data as in a, when genes selected by the BLASE gene peakedness spread method. d, BLASE result heatmap showing the same data as in a, when genes selected by the TradeSeq associationTest. e, Cell proportion estimates predicted by CIBERSORTx for the datasets described in a. f, Cell proportion estimates predicted by DWLS for the datasets described in a. g, Cell proportion estimates predicted by MeDuSA for the datasets described in a. h, Cell proportion estimates predicted by MuSiC for the datasets described in a.

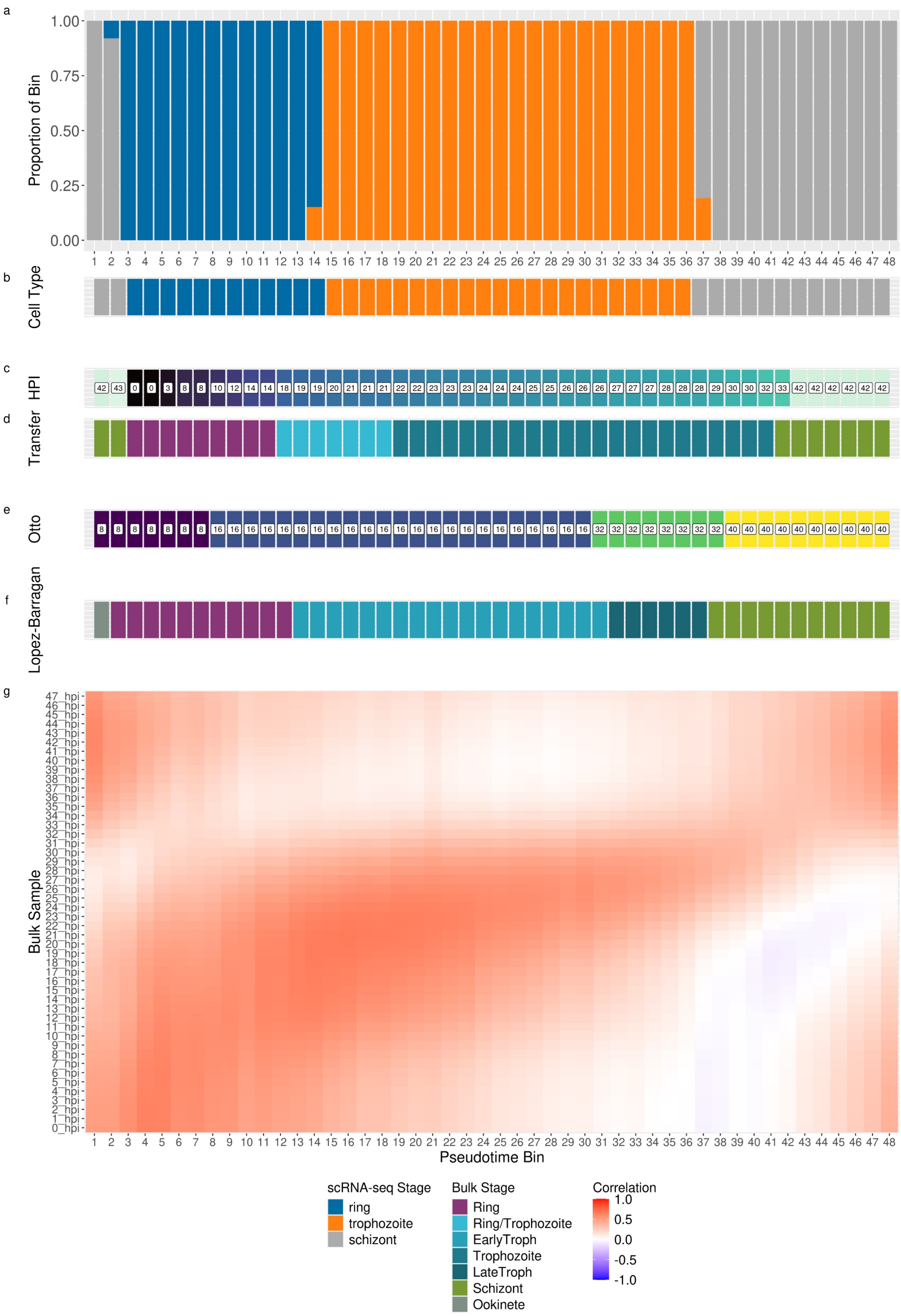

Fig. A11 Annotating *P. falciparum* scRNA-seq from bulk RNA-seq. a, Pseudotime bin populations annotated by original scRNA-seq analysis. b, Major cell type for each pseudotime bin as annotated in the original scRNA-seq (i.e. cell type with the highest proportion) c, The bulk sample (hours post infection, HPI) from Painter et al. that BLASE predicts the pseudotime bin is most likely to be from. d, The bulk sample from c, but with the expected cell types from the bulk data annotated. e, The same as c, but mapping bins to Otto et al. data. f, The same as c, but mapping bins to López-Barragán et al. data. g, The BLASE Mapping heatmap of the data used to calculate this, with 48 bins split by cell.

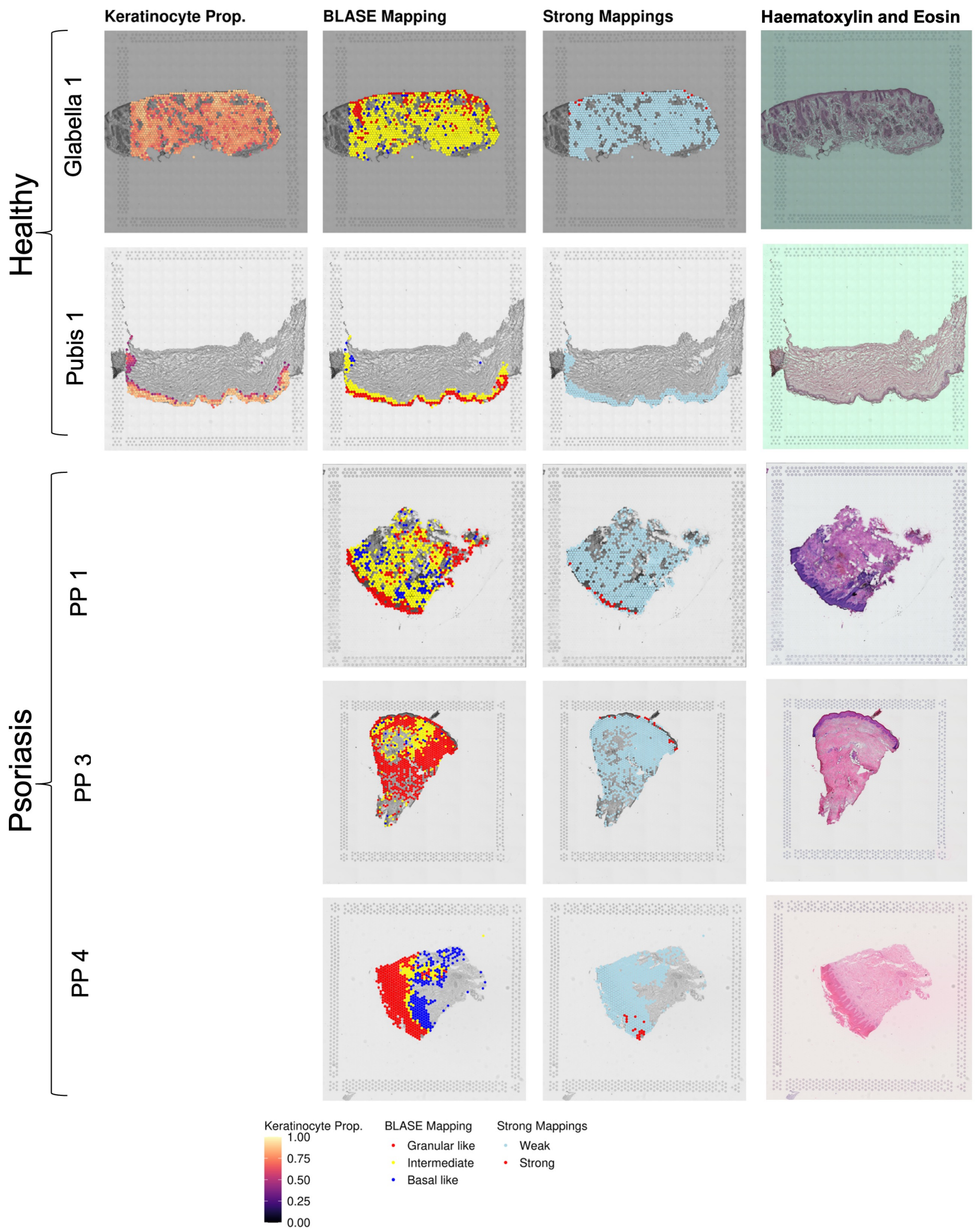

Fig. A12 Plots showing the following for a wider selection of Visium skin samples from Ganier et al. 2024 (healthy) and Ma et al. 2023 (psoriasis): Keratinocyte proportion in spot (Ganier et al. 2024 only), BLASE mappings for keratinocyte maturity in each spot, the spots with a strong BLASE mapping, and the original H&E stained samples on the Visium Slide.

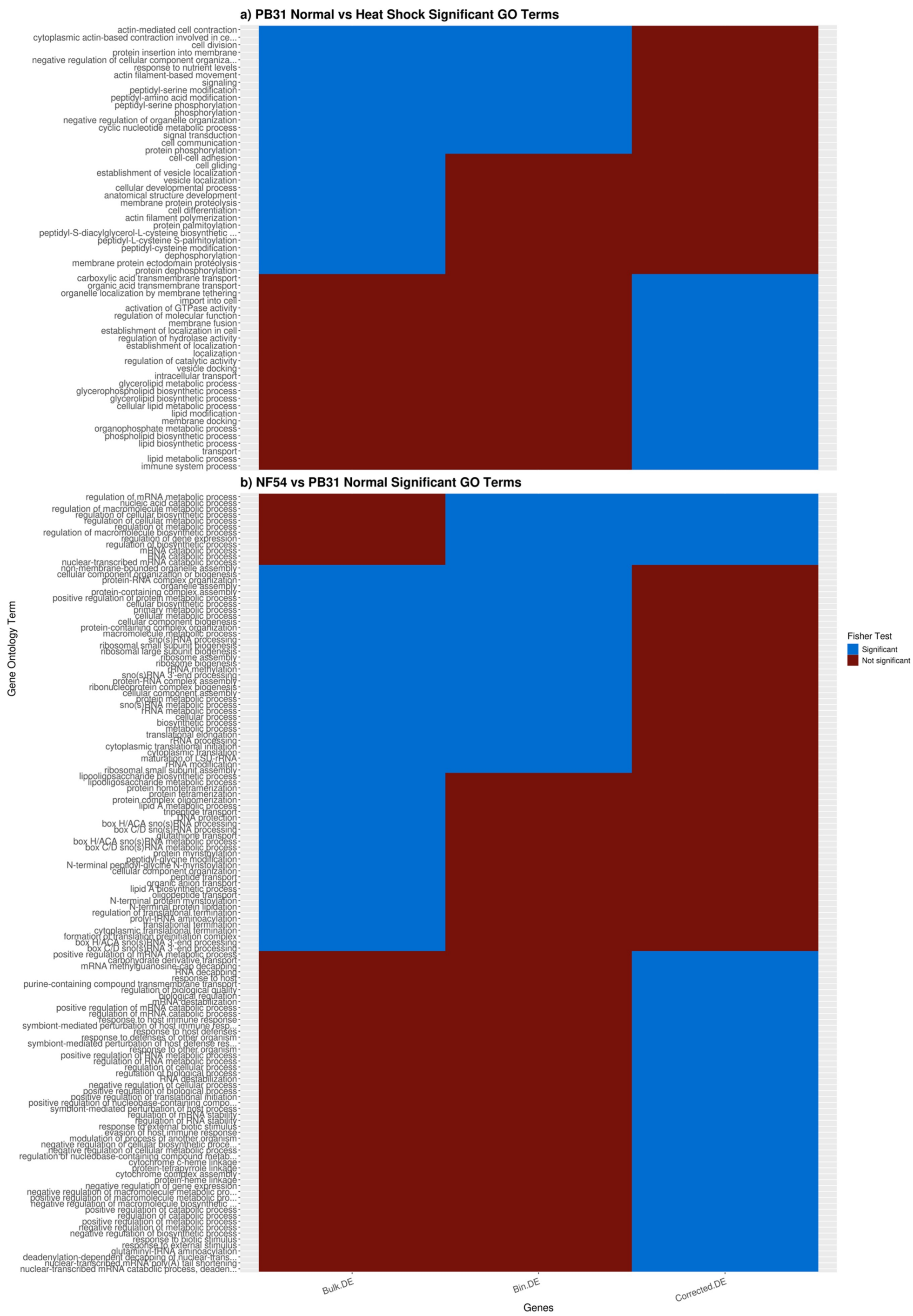

Fig. A13 Comparison of Fisher Statistic for GO enrichment analysis on gene lists from uncorrected bulk DE genes, BLASE corrected DE genes, and the list of removed genes for two comparisons: a, NF54 vs. PB31 in normal conditions, and b, PB31 in normal vs. heat shock conditions (note, no corrections were made for the NF54 normal growth conditions vs. heat shock conditions comparison, so there was no difference in GO term enrichment analysis).

Title	SC Reference	Bulk	BLASE Bins	Pseudotime Bin Assignment	BLASE Genes	Gene Selection
Simulated	Simulated	*Pseudobulked*	5	Pdt. Range	455	All genes
	”	”	5	Pdt. Range	300	Gene Peakedness
	”	”	5	Pdt. Range	30x10 (150)	Gene Peakedness Spread
	”	”	5	Pdt. Range	300	TradeSeq
Haematopoiesis	Mende et al. 2020	*Pseudobulked*	5	Pdt. Range	19178	All genes
	”	”	5	Pdt. Range	1000	Gene Peakedness
	”	”	5	Pdt. Range	50x20 (954)	Gene Peakedness Spread
	”	”	5	Pdt. Range	1000	TradeSeq
	”	”	5	Pdt. Range	100	Gene Peakedness
	”	”	5	Pdt. Range	5000	Gene Peakedness
	”	”	3	Pdt. Range	1000	Gene Peakedness
	”	”	10	Pdt. Range	1000	Gene Peakedness
Lymphoid Development	Suo et al. 2022	Casero et al. 2015	20 (19)	Cells (2 traj.)	100x10	Gene Peakedness Spread
	”	Choi et al. 2019	20 (19)	Cells (2 traj.)	100x10	Gene Peakedness Spread
Gene Selection Experiments	Howick et al. 2019	Otto et al. 2004	6	Pdt. Range	4890	All genes
	”	”	6	Pdt. Range	150	Gene Peakedness
	”	”	6	Pdt. Range	10x15 (150)	Gene Peakedness
	”	”	6	Pdt. Range	150	TradeSeq
	”	”	6	Pdt. Range	150	Cell Type Cluster Markers
	”	”	6	Pdt. Range	150	Pseudotime Bin Markers
Pf. Heat shock	Dogga et al. 2023	Zhang et al. 2021	8	Pdt. Range	30x32 (919)	Gene Peakedness Spread
Pf. Lifecycle	Dogga et al. 2023	Painter et al. 2018	6	Pdt. Range	5275	All genes
	”	”	6	Pdt. Range	800	Gene Peakedness
	”	”	6	Pdt. Range	30x32 (919)	Gene Peakedness Spread
	”	”	6	Pdt. Range	800	TradeSeq
Spatial	Wang et al. 2020	Ganier et al. 2024	3	Cells	300	Gene Peakedness
	”	Ma et al. 2023	3	Cells	300	Gene Peakedness
Medusa Reproduction	Cytotrace *C. elegans* ciliated neurons	*Pseudobulked*	5	Cells	2000	Gene Peakedness

Table A1 A comprehensive list of the BLASE hyperparameters used to generate the results presented in this paper. Pseudotime is abbreviated as “Pdt.”. Numbers in brackets indicate actual outputs, if different to target from parameter.

Title	SC Reference	Bulk	BLASE Bins	Pseudotime Bin Assignment	BLASE Genes	Gene Selection
Pf. Lifecycle	Dogga et al. 2023	Painter et al. 2018	48	Cells	30x32 (919)	Gene Peakedness Spread
	”	Otto et al. 2010	48	Cells	30x32 (919)	Gene Peakedness Spread
	”	López-Barragán et al. 2011	48	Cells	30x32 (919)	Gene Peakedness Spread

Table A2 A list of the BLASE hyperparameters used to annotate scRNA-seq psuedotime bins with known bulk RNA-seq as presented in this paper. Pseudotime is abbreviated as “Pdt.”.

	Dataset	Mean Correct Mapping Proportion
CIBERSORTx	Simulated	NA
DWLS	Simulated	0.913
MeDuSA	Simulated	0.423
MuSiC	Simulated	0.842
CIBERSORTx	Haematopoiesis	0.463
DWLS	Haematopoiesis	0.209
MeDuSA	Haematopoiesis	0.433
MuSiC	Haematopoiesis	0.750

Table A3 Mean correctly mapped proportions for other deconvolution tools, rounded to 3 decimal places.

Stage	HPI (Painter et al.)	Pseudotime Bins with > 1 cell (Dogga et al.)
Ring	0–21	1–3
Trophozoite	16–32	3–4
Schizont	33–48	2–6,1

Table A4 *P. falciparum* intraerythrocytic lifecycle stages, and their mappings in two datasets (Painter et al. 2018, Dogga et al. 2023). Ranges are inclusive. 12 pseudotime bins were used.
